# Case Report: Low-dose donor lymphocyte infusion for secondary poor graft function after haploidentical HSCT in a child with β-thalassemia

**DOI:** 10.3389/fimmu.2026.1972748

**Published:** 2026-10-23

**Authors:** Yue Li, Liping Luo, Dingzhi Heng, Chunjing Wang, Xiaohui Zhou, Xiaoying Fu, Sixi Liu

**Affiliations:** 1 Department of Hematology and Oncology, Shenzhen Children’s Hospital, Shenzhen, China; 2 Department of Laboratory Medicine, Shenzhen Children’s Hospital, Shenzhen, China

**Keywords:** donor lymphocyte infusion, haploidentical transplantation, hematopoietic stem cell transplantation, immune remodeling, pediatric, poor graft function, β-thalassemia

## Abstract

Poor graft function (PGF) is a serious complication after allogeneic hematopoietic stem cell transplantation (allo-HSCT), particularly when persistent cytopenia occurs despite complete donor chimerism. We report a 5-year-old girl with transfusion-dependent β-thalassemia major who developed refractory secondary PGF after haploidentical allo-HSCT following cytomegalovirus reactivation. Bone marrow was markedly hypocellular, and and multilineage cytopenias progressively worsened after plasma CMV DNA became undetectable and antiviral therapy was completed. Contributions from infection-related injury, antiviral-associated myelotoxicity, or endothelial injury could not be fully excluded. Multiple supportive and immunomodulatory interventions failed. Because a CD34^+^ selected stem cell boost was not feasible, the patient received intentionally low-dose donor lymphocyte infusion (DLI) on days +270 and +284. Multilineage hematopoietic recovery occurred 27 days after the first infusion, and transfusion independence was maintained for more than 14 months. Post-DLI events included manageable grade I cutaneous graft-versus-host disease and transient tachypnea of uncertain etiology. Longitudinal immune profiling showed recovery of naive T-cell compartments, contraction of effector-memory subsets, and expansion of CD8^+^CD28^-^ T cells without overt T-cell activation. This observation provides a hypothesis-generating rationale for prospective studies evaluating the efficacy and safety of low-dose DLI in pediatric secondary PGF.

## 1 Introduction

Poor graft function (PGF) is characterized by persistent cytopenia after allo-HSCT in the presence of complete donor chimerism. Its reported incidence is approximately 5%-27%, depending on transplant platform, disease setting, and diagnostic criteria (1). PGF is associated with transfusion dependence, infection risk, impaired quality of life, and increased non-relapse morbidity.

The pathogenesis of PGF is multifactorial and remains incompletely understood. Viral reactivation, myelotoxic drugs, inflammatory injury, and impairment of the bone marrow microenvironment have all been implicated (2–5). Increasing evidence indicates that aberrant T-cell responses, type 1 inflammatory signaling, and impaired mesenchymal stem cells (MSC) support may create a bone marrow microenvironment that is unfavorable for hematopoietic recovery (5–7).

Current therapeutic approaches include hematopoietic growth factors, thrombopoietin receptor agonists, MSC, immunomodulatory therapy, and CD34^+^ selected stem cell boost from the original donor (5). Retrospective studies have reported high hematologic response rates following CD34^+^-selected stem cell boost, although outcomes vary across patient populations and transplant settings, and access to cell selection may be limited (8, 9). Donor lymphocyte infusion (DLI) is generally used to enhance graft-versus-leukemia activity or donor chimerism; however, donor T cells may also influence engraftment and immune homeostasis within the bone marrow microenvironment (10). Evidence for DLI in pediatric PGF and non-malignant disorders remains scarce. Here, we describe a child with β-thalassemia major who developed refractory secondary PGF after haploidentical allo-HSCT and achieved durable hematopoietic recovery after intentionally low-dose DLI, accompanied by longitudinal immune remodeling.

## 2 Case presentation

A 5-year-old girl with transfusion-dependent β-thalassemia major (β0/β0 genotype), diagnosed at 9 months of age, underwent haploidentical peripheral blood stem cell transplantation from her father. Before transplantation, she received regular red blood cell transfusions and oral iron chelation therapy, with a pre-transplant serum ferritin level of approximately 3, 400 ng/mL. The spleen was normal in size, and the liver edge was palpable 2 cm below the right costal margin. Minor ABO incompatibility was present, and donor-specific anti-HLA antibodies were negative.

Pre-transplant immunosuppression consisted of hydroxyurea and azathioprine. Myeloablative conditioning comprised cyclophosphamide at 50 mg/kg/day on days −8 and −7; fludarabine at 40 mg/m²/day on days −6 to −2; busulfan at 3.2 mg/kg/day in four divided doses on days −6 to −4; thiotepa at 10 mg/kg on day −3; and rabbit anti-thymocyte globulin (Thymoglobuline; Sanofi) at 1 mg/kg/day on days −3 to −1. The measured busulfan steady-state concentration was 717.5 ng/mL (target, 600–800 ng/mL). The infused graft contained 20 × 10^8^ nucleated cells/kg and 19 × 10^6^ CD34^+^ cells/kg.

Graft-versus-host disease (GVHD) prophylaxis consisted of post-transplant cyclophosphamide at 50 mg/kg/day on days +3 and +4, tacrolimus and short-course mycophenolate mofetil (MMF). MMF was discontinued on day +23 because of human metapneumovirus infection. Neutrophil and platelet engraftment were achieved on days +16 and +24. Donor chimerism on day +18 was 99.5% - 99.9% in whole blood and T-cell, B-cell, and natural killer (NK)-cell fractions; subsequent chimerism testing was limited to whole blood and T cells.

Approximately 2 months after allo-HSCT, the patient developed cytomegalovirus (CMV) DNAemia, followed by progressive pancytopenia. The highest documented plasma CMV DNA level was 1.01 × 10³ copies/mL. Ganciclovir was administered for 2 weeks; CMV DNA first became undetectable 7 days after initial detection, with negativity confirmed on two consecutive tests. Cytopenias progressively worsened after virologic clearance and treatment completion.

Bone marrow examination on day + 142 showed marked hypocellularity (<5%), while whole-blood and T-cell donor chimerism remained >99% during PGF (**Supplementary Table 1**). These findings, together with persistent multilineage cytopenias after initial engraftment, supported a diagnosis of secondary PGF. Ultrasonography during PGF showed no splenomegaly. No clinical organ GVHD was evident before DLI; antiglobulin and antiplatelet antibody tests were negative. Subsequent weekly plasma CMV and Epstein–Barr virus (EBV) DNA tests were negative, as was parvovirus B19 DNA testing on day +142. Neither bone marrow CMV DNA testing nor blood DNA testing for adenovirus or human herpesvirus 6B (HHV-6B) was performed.

Respiratory syncytial virus (RSV) pneumonia with a small pericardial effusion developed on day +151 and was treated with antibacterial agents and ribavirin. Dyspnea and hypoxemia on day +164 prompted adjunctive methylprednisolone at 2 mg/kg/day for 3 days. Bronchoalveolar lavage (BAL) was performed on day +165 to investigate respiratory deterioration; sequencing detected RSV type A, *Pseudomonas aeruginosa, Stenotrophom*onas maltophilia, and HHV-6B (17 reads; clinical significance uncertain). Antibacterial therapy was adjusted accordingly. Respiratory symptoms resolved after these combined interventions, with radiographic improvement by day +184.

The pericardial effusion became large by day +184 and progressed on day +190 despite methylprednisolone and one dose of tocilizumab. Tachycardia and oliguria raised concern for cardiac tamponade. Cardiac troponin I and lactate dehydrogenase were elevated, but blood pressure and renal function were normal. No schistocytes were detected, urine protein testing was negative, and soluble C5b-9 was not measured. Suspected calcineurin inhibitor-associated endothelial injury prompted replacement of tacrolimus with low-dose MMF on day +190. With methylprednisolone and diuretics continued, only a small effusion remained by day +229. Sirolimus was initiated on day +243, and the pre-DLI methylprednisolone course was discontinued on day +246. Transplantation-associated thrombotic microangiopathy (TA-TMA) was not confirmed; infection-related injury, antiviral-associated myelotoxicity, and endothelial injury could not be excluded as contributors to the cytopenias.

Interventions before DLI included methylprednisolone, rituximab, intravenous immunoglobulin, MSC infusion, granulocyte colony-stimulating factor, thrombopoietin receptor agonists, and low-dose decitabine, without sustained hematopoietic recovery. The patient remained transfusion dependent. Because financial constraints precluded a CD34-selected stem cell boost, low-dose DLI was administered without additional conditioning at 7.5 × 10^4^ CD3^+^ T cells/kg on day +270 and 1.5 × 10^5^ CD3^+^ T cells/kg on day +284. MMF had been discontinued before the first DLI, whereas sirolimus continued for GVHD prophylaxis (**Figure 1**).

**Figure 1:**
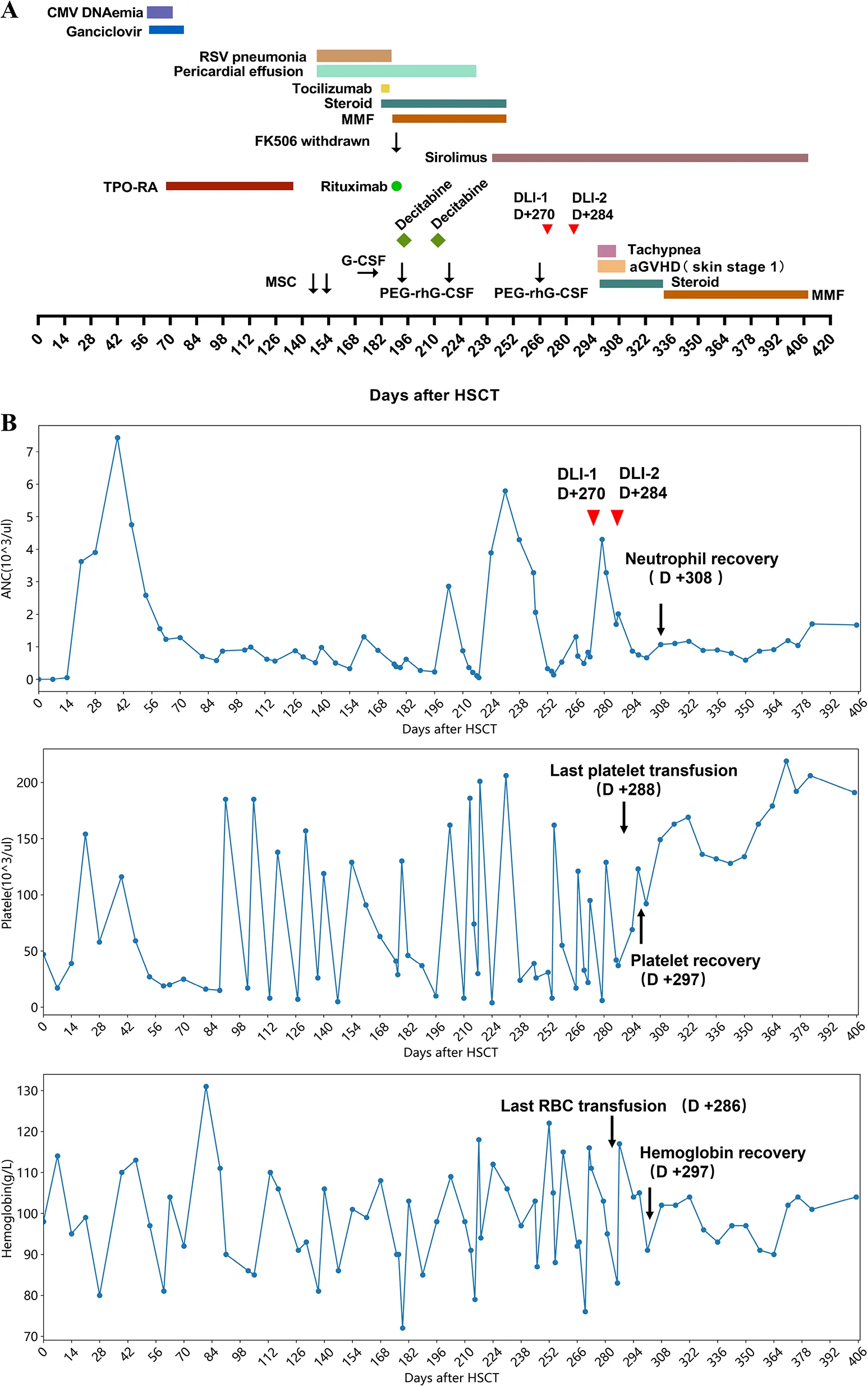
Clinical course and hematologic recovery after HSCT and low-dose DLI. **(A)** Treatment timeline. **(B)** Serial absolute neutrophil count, platelet count, and hemoglobin levels.

Erythroid and platelet recovery occurred 27 days after the first DLI, followed by neutrophil recovery at 38 days. One month after DLI, the patient developed grade I cutaneous GVHD and responded to topical corticosteroids. Mild tachypnea occurred on day +300, with an unremarkable chest CT and no other documented symptoms of respiratory infection. Oral methylprednisolone was initiated empirically at 0.5 mg/kg/day, primarily for tachypnea. The respiratory rate normalized, and tapering began on day +306. The respiratory episode remained of uncertain etiology, an infectious cause could not be conclusively excluded. Declining granulocyte and platelet counts and hemoglobin concentration prompted methylprednisolone withdrawal and MMF reintroduction (15 mg/kg/day) on day +329. Blood counts subsequently improved without recurrence of tachypnea or cutaneous GVHD. Transfusion independence was maintained for more than 14 months.

Peripheral blood lymphocyte subsets were analyzed by flow cytometry on day +267 before DLI and on days + 302, + 554 afterward Before DLI, the patient showed an immune profile characterized by increased effector-memory T-cell subsets, increased inducible CD4+ regulatory T cells, and reduced naive T-cell compartments. On day +302, CD28^−^ cell proportions increased within both CD4^+^ and CD8^+^ T-cell populations, whereas the proportions of phenotypically defined Tregs decreased. On day +554, the proportions of naive-phenotype CD4^+^ and CD8^+^ T cells were higher than at baseline. CD4^+^ central memory and effector memory T-cell proportions were lower, whereas CD8^+^ memory T-cell subsets showed divergent changes. Total Treg proportion below baseline at 10.10% of CD4^+^ T cells, and the CD45RA^+^ fraction within the Treg population was higher than at baseline (**Figure 2**; **Supplementary Table 3**).

**Figure 2:**
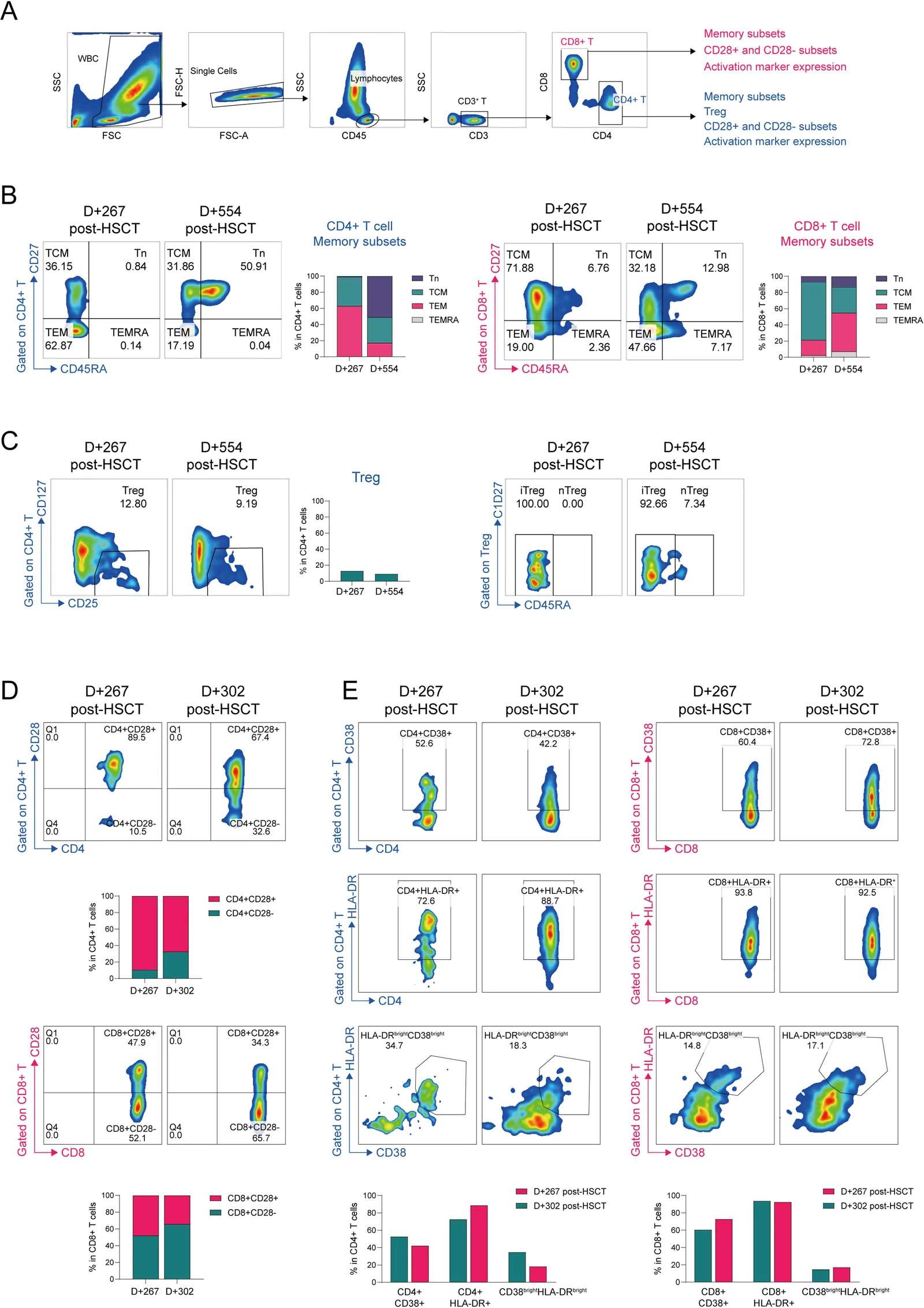
Peripheral blood T-cell subsets and activation-marker expression before and after DLI. **(A)** Gating strategy for CD4^+^ and CD8^+^ T-cell analyses. **(B)** CD4^+^ and CD8^+^ T-cell memory subsets on days +267 and +554. **(C)** Regulatory T cells **(Tregs)** and their CD45RA-defined subsets on days +267 and +554. **(D)** CD28^+^ and CD28^−^ subsets within CD4^+^ and CD8^+^ T cells on days +267 and +302. **(E)** CD38^+^, HLA-DR^+^, and CD38^bright^HLA-DR^bright^ T-cell frequencies on days +267 and +302. Detailed gating criteria and subset definitions are provided in the Supplementary Methods. Percentages are relative to the corresponding CD4^+^ or CD8^+^ population in panels **(B, D, E)**; CD4^+^ T cells for total Tregs; and total Tregs for CD45RA-defined fractions. All sampling days are relative to HSCT.

Laboratory-reported absolute lymphocyte counts increased from 388 cells/μL on day +267 to 2, 688 cells/μL on day +554, with increases in CD3^+^, CD4^+^, and CD8^+^ T-cell counts (**Supplementary Table 2**). On day +302, the complete blood count (CBC) absolute lymphocyte count was 550 cells/μL, but absolute major-subset counts were unavailable.

## 3 Materials and methods

Peripheral blood samples were collected using EDTA-2K as an anticoagulant to evaluate the patient’s immune profiling before DLI on day +267 and after DLI on days +302 and +554. All sampling days refer to days after HSCT. The following antibodies were utilized: anti-human-CD45 V500, CD28-PE, CD4-PE-Cy7, CD3-APC, CD8-PerCP, CD38-FITC, HLA-DR-APC-Cy7, CD45-V500, anti-human-CD3-APC-Cy7, PE-Cy7-CD27, CD4-V450, CD127-PE, CD25-APC, CD45RA-FITC for immune evaluation. Antibodies and specimens were thoroughly mixed and incubated at room temperature for 15 minutes, protected from light. Subsequently, 1 mL of lysis buffer was added for red blood cell removal, followed by a 15-minute incubation away from light. The samples were washed twice with 2 mL of PBS and centrifuged at 1500 rpm for 5 minutes. After removing the supernatant, 300 μL of PBS was added in preparation for testing. Analysis was performed using a FACS Canto II cytometer (BD Biosciences, San Jose, CA, USA). All antibodies were obtained from Becton Dickinson (Mountain View, CA, USA) and Beckman Coulter (Brea, CA, USA), and data analysis was conducted using FlowJo 10.0.

Clinical lymphocyte-subset results were reported separately, with available absolute major-subset counts on days +267 and +554 and the complete blood count (CBC) absolute lymphocyte count on day +302 (**Supplementary Table 2**). Frequencies were expressed relative to the specified parent populations. Gating procedures, gating strategies, subset definitions, and percentage denominators are provided in the **Supplementary Methods**; clinical-report frequencies are presented in **Supplementary Table 3**.

## 4 Discussion

This case documents sustained hematopoietic recovery following low-dose DLI without additional conditioning in a child with refractory secondary PGF after haploidentical HSCT for β-thalassemia. Multiple prior interventions had not produced sustained recovery, whereas transfusion independence was maintained for more than 14 months after DLI.

The relatively high infused CD34^+^ cell dose and successful initial engraftment argue against insufficient initial cell numbers as the principal explanation for secondary PGF. Persistent cytopenias despite sustained complete donor chimerism are consistent with impaired donor-derived hematopoiesis, but do not identify whether stem/progenitor cells, their supporting microenvironment, or both were affected (3–5). The preceding CMV episode may have contributed to the onset of marrow dysfunction. Although cytopenias worsened after clearance of plasma CMV DNAemia and completion of antiviral therapy, contributions from infection-related injury or ganciclovir-associated myelotoxicity could not be excluded. The absence of marrow CMV testing and incomplete assessment of endothelial injury further limit etiologic interpretation.

CD34-selected stem cell boost for PGF is supported mainly by retrospective studies (8, 11, 12). A meta-analysis of seven studies involving 209 adults reported pooled complete and overall response rates of 72% and 80%, respectively (8). Pediatric series have also reported hematopoietic recovery, although differences in patient populations and transplant settings limit extrapolation to children undergoing haploidentical HSCT for non-malignant disorders (9). However, active infection, persistent inflammatory burden and an unfavorable marrow microenvironment are associated with treatment failure (11, 12). Financial constraints precluded a CD34-selected boost in our patient.

Studies in other transplantation settings suggest that T-cell subsets influence progenitor homing and engraftment, while memory CD8^+^ T cells and regulatory T cells can support stem cell maintenance or marrow niche function in experimental models (13–17). These findings provide a biological rationale for investigating DLI in PGF.

Published DLI experience for PGF is limited and heterogeneous (**Table 1**). Hamada et al. described a 3-year-old boy with hyper-IgM syndrome and secondary PGF after unrelated bone marrow transplantation (10). A single unconditioned domino DLI of 1 × 10^6^ CD3^+^ cells/kg was administered using cells from his HLA-identical elder brother, who had previously received a transplant from the same unrelated donor. Pancytopenia improved after approximately one month, with transfusion independence during two years of follow-up; grade I cutaneous GVHD responded to topical corticosteroids. This donor configuration differs from direct parental haploidentical DLI.

**Table 1:** Published DLI-based approaches for PGF and comparison with the present case.

Report	Patients and transplant setting	PGF diagnosis	Treatment strategy and cell product	Hematologic outcomes and GVHD
Hamada et al. 2021 (10)	One 3-year-old boy with hyper-IgM syndrome; HLA-C1 allele-mismatched unrelated BMT.	Secondary PGF; progressive pancytopenia from day +134; marrow cellularity <10%; full donor chimerism.	One domino DLI on day +198, without conditioning: 1 × 10^6^ CD3^+^ cells/kg. Source: HLA-identical elder brother previously transplanted from the same unrelated donor. CD34^+^ content not reported.	Pancytopenia improved after approximately 1 month; subsequent transfusion independence reported during 2-year follow-up. Grade I cutaneous GVHD on day +212, responsive to topical corticosteroids.
Yuan et al. 2026 (18)	10 adults; median age 48.5 years (24–58). AML 7; ALL 1; PMF 1; T-LBL 1; haplo-HSCT.	Primary PGF: cytopenia >2 weeks after day +28; donor chimerism >95%; included single-lineage cytopenias.	Decitabine 7 mg/m²/day ×3; DLI + eltrombopag 50–150 mg/day from regimen day 5. Median CD3^+^: 4.91×10^7^/kg; CD34^+^: 0.44×10^6^/kg. Cyclosporine + short-course methotrexate.	CR 9/10; PR 1/10; responses maintained in 8 survivors. Acute GVHD: grade I, 2; grade III, 1. Mild chronic GVHD: 1.
Present case	One 5-year-old girl with transfusion-dependent β-thalassemia; paternal haploidentical peripheral blood HSCT.	Secondary PGF after initial engraftment; persistent multilineage cytopenias; marrow cellularity <5%; donor chimerism >95%.	Two DLIs without additional conditioning: 7.5 × 10^4^ CD3^+^ cells/kg on day +270 and 1.5 × 10^5^/kg on day +284; sirolimus continued. CD34^+^ content not reported.	Recovery within 27 days of first DLI; transfusion independence >14 months. Grade I cutaneous GVHD and mild tachypnea.

ALL, acute lymphoblastic leukemia; AML, acute myeloid leukemia; BMT, bone marrow transplantation; CR, complete response; DLI, donor lymphocyte infusion; GVHD, graft-versus-host disease; HSCT, hematopoietic stem cell transplantation; PGF, poor graft function; PMF, primary myelofibrosis; PR, partial response; T-LBL, T-lymphoblastic lymphoma.

Yuan et al. reported nine complete and one partial response in 10 adults with primary PGF treated with decitabine, DLI, and eltrombopag after haploidentical HSCT (18). The median infused doses were 4.91 × 10^7^ CD3^+^ cells/kg and 0.44 × 10^6^ CD34^+^ cells/kg; one patient developed grade III acute GVHD. Concurrent drugs and hematopoietic progenitors prevent attribution of benefit to lymphocytes alone. Differences in PGF phenotype, donor configuration, and accompanying treatment limit direct comparisons with our case.

The intentionally low DLI dose was selected for several clinical reasons in this patient. First, the non-malignant indication required no graft-versus-leukemia effect, and the aim was to explore a potential immunomodulatory benefit while limiting toxicity. Second, the haploidentical setting and the preceding CMV episode prompted caution about GVHD. Finally, the patient remained profoundly cytopenic and clinically fragile further favored low doses.

Following DLI, grade I cutaneous acute GVHD responded to topical corticosteroids, whereas systemic methylprednisolone was given empirically for accompanying tachypnea. Improvement did not establish the cause of the respiratory tachypnea, and occult infection could not be excluded. Interestingly, transient declines in blood counts occurred during systemic corticosteroid therapy but stabilized after corticosteroid withdrawal and reintroduction of low-dose MMF. Although causality cannot be inferred from a single case, this temporal pattern raises the possibility that the type or intensity of post-DLI immunosuppression may influence hematopoietic recovery. This observation is consistent with the concept that broad immunosuppression can control alloimmune inflammation but may also affect immune mechanisms involved in hematopoietic restoration within the bone marrow microenvironment (19). Accordingly, refractory PGF may require therapeutic strategies that balance immune modulation with restoration of hematopoietic function and immune homeostasis (5).

The longitudinal immune profile provides an exploratory view of immune reconstitution. At early follow-up, CD8^+^CD28^−^ frequencies increased. At later follow-up, higher naive-phenotype T-cell proportions were consistent with naive T-cell reconstitution, accompanied by lower CD4^+^ effector-memory frequencies and divergent CD8^+^ memory changes. Selected CD8^+^CD28^−^ populations have been associated with immunoregulatory activity or suppressive properties in some settings (20, 21). Together, these findings are consistent with immune remodeling toward a more homeostatic T-cell composition rather than broad immune activation. The available absolute counts documented later increases in major lymphocyte populations, but complete paired counts were unavailable for detailed T-cell subsets. Changes in subset frequencies may therefore reflect shifts in the size or composition of the parent population rather than corresponding absolute expansion or depletion. These data also cannot determine whether the immune changes contributed to hematopoietic recovery or accompanied it. A role for DLI-mediated immune remodeling therefore remains a working hypothesis.

This single case cannot separate DLI effects from delayed responses to prior therapies, changes in immunosuppression, or spontaneous recovery. Functional assays and direct characterization of marrow immune and stromal compartments were not performed. Longer follow-up is required to evaluate durability and late complications, including chronic GVHD. Prospective pediatric studies integrating longitudinal subset frequencies, contemporaneous absolute counts, and functional assessments are warranted to evaluate efficacy, safety, and mechanisms and to inform DLI dosing, timing, concomitant immunosuppression, and patient selection.

## 5 Conclusions

This case documents sustained hematopoietic recovery following low-dose DLI without additional conditioning in a child with refractory secondary PGF after haploidentical HSCT for β-thalassemia. The clinical and immune observations are hypothesis-generating and warrant prospective studies to evaluate efficacy, safety, and the relationship between immune reconstitution and hematopoietic recovery.

## Data Availability

The original contributions presented in the study are included in the article/**Supplementary Material**. Further inquiries can be directed to the corresponding authors.
