# Supplementary material for: Case Report: Low-dose donor lymphocyte infusion for secondary poor graft function after haploidentical HSCT in a child with β-thalassemia
Source: Front Immunol. 2026 Oct 23;17:1972748. doi: 10.3389/fimmu.2026.1972748 (PMC13739708; doi:10.3389/fimmu.2026.1972748)
Supplement: Supplementary file 1 [file SupplementaryFile1.docx]

# **Supplementary Table S1. Donor chimerism at key post-transplant assessments**

| **Assessment** | **Day after HSCT** | **Donor chimerism (%)** | | | |
| --- | --- | --- | --- | --- | --- |
|  |  | **Whole blood** | **T cells** | **B cells** | **NK cells** |
| Initial assessment | +18 | 99.6 | 99.7 | 99.9 | 99.5 |
| CMV viremia | +60 | 99.6 | 99.8 | Not assessed | Not assessed |
| PGF evaluation | +80 | 99.7 | 99.5 | Not assessed | Not assessed |
| Before DLI | +267 | 99.8 | 99.8 | Not assessed | Not assessed |
| After DLI | +329 | 99.7 | 99.8 | Not assessed | Not assessed |

All values represent donor chimerism percentages in the specified specimen or cell fraction. "Not assessed" indicates that the corresponding cell fraction was not tested at that time point.

Abbreviations: HSCT, hematopoietic stem cell transplantation; CMV, cytomegalovirus; PGF, poor graft function; DLI, donor lymphocyte infusion; NK, natural killer.

# Supplementary Methods

**Flow cytometric lymphocyte-subset analysis**

**Gating and subset definitions**

Debris and doublets were excluded using FSC-A/SSC-A and FSC-A/FSC-H, respectively. CD3⁺ T cells were identified within CD45⁺ cells and separated into CD4⁺ and CD8⁺ populations (Figure 2A). Within each population, memory subsets were defined by CD45RA and CD27 expression: naive (Tn), CD45RA⁺CD27⁺; central memory (TCM), CD45RA⁻CD27⁺; effector memory (TEM), CD45RA⁻CD27⁻; and terminally differentiated effector memory (TEMRA), CD45RA⁺CD27⁻. Regulatory T cells (Tregs) were phenotypically defined as CD4⁺CD25⁺CD127^dim^ cells and further divided into CD45RA⁺ and CD45RA⁻ fractions. CD28⁺ and CD28⁻ fractions were assessed within both CD4⁺ and CD8⁺ T-cell populations. Activation-marker analyses included CD38⁺, HLA-DR⁺, and CD38^bright^HLA-DR^bright^ populations. The double-bright gate in Figure 2 identifies cells with high expression of both markers.

**Percentage denominators and absolute counts**

Routine clinical lymphocyte-subset panels were also performed by flow cytometry. Major lymphocyte-lineage percentages were expressed relative to total lymphocytes. Memory, CD28-defined, and activation-marker frequencies were expressed relative to the corresponding CD4⁺ or CD8⁺ population, except that CD3⁺HLA-DR⁺ frequencies were expressed relative to CD3⁺ T cells. Total Treg frequencies were expressed relative to CD4⁺ T cells. CD45RA-defined Treg fractions were expressed relative to total Tregs in Figure 2 and to CD4⁺ T cells in the clinical reports (Supplementary Table S3).

Laboratory-reported absolute counts of major lymphocyte subsets were available on days +267 and +554. On day +302, only the complete blood count (CBC) absolute lymphocyte count was available. These values are presented in Supplementary Table S2. Detailed absolute subset counts were not reconstructed by combining CBC results with flow cytometric percentages. Complete matched absolute counts were not available for all detailed subsets and sampling time points.

**Supplementary Table S2. Flow cytometric lymphocyte-subset results on days +267 and +554 after HSCT.**

| **Population** | **D+267** | | **D+554** | |
| --- | --- | --- | --- | --- |
|  | **% of lymphocytes** | **Reported count (cells/µL)** | **% of lymphocytes** | **Reported count (cells/µL)** |
| Total lymphocytes | — | 388 | — | 2,688 |
| CD3⁺ T cells | 77.12 | 299 | 73.30 | 1,970 |
| CD4⁺ T cells | 14.00 | 55 | 23.45 | 630 |
| CD8⁺ T cells | 60.92 | 239 | 48.22 | 1,295 |
| B cells | 0.08 | 0 | 7.43 | 200 |
| NK cells | 22.12 | 85 | 16.63 | 447 |

All percentages use total lymphocytes as the denominator. The dash indicates that a percentage is not applicable.

D+ indicates days after HSCT. Absolute major-subset counts were unavailable for day +302, when the complete blood count absolute lymphocyte count was 550 cells/µL.

Abbreviations: HSCT, hematopoietic stem cell transplantation; NK, natural killer; Treg, regulatory T cell.

**Supplementary Table S3. Reported T-cell subset frequencies and their parent populations.**

**A. Memory and regulatory T-cell panels**

| **Population** | **Percentage denominator** | **D+267 (%)** | **D+302 (%)** | **D+554 (%)** |
| --- | --- | --- | --- | --- |
| CD3^+^ T cells | Lymphocytes | 59.70 | 78.30 | 72.50 |
| CD4^+^ T cells | Lymphocytes | 13.30 | 27.70 | 23.00 |
| CD8^+^ T cells | Lymphocytes | 44.80 | 49.40 | 47.40 |
| CD4^+^ naive T cells | CD4^+^ T cells | 0.90 | 0.30 | 45.50 |
| CD4^+^ central memory T cells | CD4^+^ T cells | 37.70 | 21.80 | 36.60 |
| CD4^+^ effector memory T cells | CD4^+^ T cells | 60.30 | 77.60 | 17.70 |
| CD4^+^ TEMRA cells | CD4^+^ T cells | 1.10 | 0.30 | 0.20 |
| CD8^+^ naive T cells | CD8^+^ T cells | 4.60 | 3.40 | 13.10 |
| CD8^+^ central-memory T cells | CD8^+^ T cells | 73.40 | 69.50 | 31.30 |
| CD8^+^ effector-memory T cells | CD8^+^ T cells | 20.20 | 25.70 | 47.60 |
| CD8^+^ TEMRA cells | CD8^+^ T cells | 1.80 | 1.40 | 8.00 |
| Total phenotypic Tregs | CD4^+^ T cells | 13.10 | 6.00 | 10.10 |
| CD45RA^+^ Tregs | CD4^+^ T cells | 0.40 | 0.05 | 1.37 |
| CD45RA^-^ Tregs | CD4^+^ T cells | 12.35 | 5.31 | 8.76 |

T-cell memory subsets were defined by CD45RA and CD27: naive, CD45RA⁺CD27⁺; central memory, CD45RA⁻CD27⁺; effector memory, CD45RA⁻CD27⁻; and TEMRA, CD45RA⁺CD27⁻.

Tregs were phenotypically defined in the clinical reports as CD4⁺CD25⁺CD127^dim^ cells. The clinical labels “natural” and “induced” are represented here by the measured CD45RA phenotype, without assigning developmental origin or suppressive function.

**Supplementary Table S3. Continued.**

**B. Activation and CD28-expression panels on days +267 and +302**

| **Population** | **Percentage denominator** | **D+267 (%)** | **D+302 (%)** |
| --- | --- | --- | --- |
| CD3⁺ T cells | Lymphocytes | 58.41 | 65.11 |
| CD4⁺ T cells | Lymphocytes | 20.92 | 19.35 |
| CD8⁺ T cells | Lymphocytes | 45.95 | 45.62 |
| CD3⁺HLA-DR⁺ cells | CD3⁺ T cells | 43.43 | 50.63 |
| CD4⁺CD28⁺ cells | CD4⁺ T cells | 85.58 | 81.45 |
| CD4⁺CD38⁺ cells | CD4⁺ T cells | 39.42 | 28.96 |
| CD4⁺HLA-DR⁺ cells | CD4⁺ T cells | 57.69 | 66.67 |
| CD4⁺CD38⁺HLA-DR⁺ cells | CD4⁺ T cells | 36.54 | 11.61 |
| CD8⁺CD28⁺ cells | CD8⁺ T cells | 68.93 | 38.44 |
| CD8⁺CD28⁻ cells | CD8⁺ T cells | 31.00 | 62.00 |
| CD8⁺CD38⁺ cells | CD8⁺ T cells | 35.05 | 46.50 |
| CD8⁺HLA-DR⁺ cells | CD8⁺ T cells | 52.34 | 51.47 |
| CD8⁺CD38⁺HLA-DR⁺ cells | CD8⁺ T cells | 19.86 | 9.77 |

Abbreviations: D+, days after HSCT; Treg, regulatory T cell; TEMRA, terminally differentiated effector-memory T cell re-expressing CD45RA. All percentages use the explicitly stated parent population.
